# Use of an Electronic Patient Portal Among the Chronically Ill: An Observational Study

**DOI:** 10.2196/jmir.3722

**Published:** 2014-12-08

**Authors:** Iiris Riippa, Miika Linna, Ilona Rönkkö, Virpi Kröger

**Affiliations:** ^1^Aalto UniversityEspooFinland; ^2^Hämeenlinnan Terveyspalvelut Public UtilityHämeenlinnaFinland

**Keywords:** chronic illness, patient portal, service channel, user profile

## Abstract

**Background:**

Electronic patient portals may enhance effective interaction between the patient and the health care provider. To grasp the full potential of patient portals, health care providers need more knowledge on which patient groups prefer electronic services and how patients should be served through this channel.

**Objective:**

The objective of this study was to assess how chronically ill patients’ state of health, comorbidities, and previous care are associated with their adoption and use of a patient portal.

**Methods:**

A total of 222 chronically ill patients, who were offered access to a patient portal with their health records and secure messaging with care professionals, were included in the study. Differences in the characteristics of non-users, viewers, and interactive users of the patient portal were analyzed before access to the portal. Patients’ age, gender, diagnoses, levels of the relevant physiological measurements, health care contacts, and received physiological measurements were collected from the care provider’s electronic health record. In addition, patient-reported health and patient activation were assessed by a survey.

**Results:**

Despite the broad range of measures used to indicate the patients’ state of health, the portal user groups differed only in their recorded diagnosis for hypertension, which was most common in the non-user group. However, there were significant differences in the amount of care received during the year before access to the portal. The non-user group had more nurse visits and more measurements of relevant physiological outcomes than viewers and interactive users. They also had fewer referrals to specialized care during the year before access to the portal than the two other groups. The viewers and the interactive users differed from each other significantly in the number of nurse calls received, the interactive users having more calls than the viewers. No significant differences in age, gender, or patient activation were detected between the user groups.

**Conclusions:**

Previous care received by the patient is an important predictor for the use of a patient portal. In a group of patients with a similar disease burden, demand for different types of health services and preferences related to the service channel seem to contribute to the choice to use the patient portal. Further research on patient portal functionalities and their potential to meet patient needs by complementing or substituting for traditional health care services is suggested.

## Introduction

The electronic patient portal is an increasingly popular channel for health care providers to offer information to and interact with their patients. Typically, a patient portal includes patients’ own health records, drawn from the care provider’s electronic health records (EHR), and the possibility of interacting with the provider through secure messaging in non-acute matters and to request repeat prescriptions [1]. More advanced portals may also offer personally tailored health information and social functionalities that enable peer support from other patients [2].

The potential benefits of patient portals include the empowerment and activation of patients in the management of their own health through increased access to related information [2,3]. In addition, interaction through a patient portal may improve the efficiency of care by replacing some of the service contacts previously performed in person or via phone calls, which are thus bound to time and often to place [4].

The suggested benefits of the patient portals may, however, be unequally distributed among patients, owing to differing interest in, access to, or ability to use the service [5,6]. To grasp the full potential of patient portals and to avoid unequal access to care, health care providers need to know which of their patients may be served by this means, and how. This understanding may be acquired by identifying differences in characteristics between portal adopters and non-adopters and between users and non-users of specific functionalities. Previous studies have reported disparities in patient use of health information technology mostly by sociodemographic factors [7]. As the reported associations between sociodemographic factors and use of health information technology have been contradictory [7], other patient factors may explain the differential use of specific services offered by means of health information technology. In studies on patient portal use, little attention has been paid to patients’ prior health care consumption patterns, that is, care received by the patients prior to portal access.

This study focused on disparities in patient portal use by patients’ state of health and previous care received. As the adoption of a patient portal requires some level of patient participation, we also tested for the relationship between patient activation (knowledge, skills, and confidence in managing one’s condition) and portal use. In addition, the associations of age and gender with portal use were assessed.

In chronic care, “frequent interactions with the provider [are] required and sustained effort [is] needed of the patient to manage his/her disease” [8]. It is therefore suggested that the chronically ill are likely to benefit from the use of an electronic patient portal [8]. Consequently, the present study was conducted among the chronically ill.

## Methods

### Study Setting, Participants, and Description of the Portal

The study setting was Finnish public primary care in a medium-sized Finnish town, Hämeenlinna, with c. 68,000 inhabitants and 10 health centers. In Finland, health services are mainly funded by municipalities from tax revenue. Municipalities are responsible for providing all necessary health services to their residents. Typically, primary care services are provided locally in health centers, whereas hospital districts formed by municipalities are responsible for arranging specialized medical care that is centralized in larger towns. Follow-up and maintenance of the chronically ill are one of the main tasks of public primary care in Finland.

The study group consisted of chronically ill, existing customers of the care provider, who had participated in a controlled before/after study reported elsewhere [9]. The eligibility criteria for the participants were: (1) age of at least 18 years, (2) has at least two treatable health conditions assessed by a health professional, (3) has bank identifiers for electronic identification and access to the Internet, and (4) is willing and able, both according to themselves and to a health care professional, to engage in using the portal.

**Figure 1:**
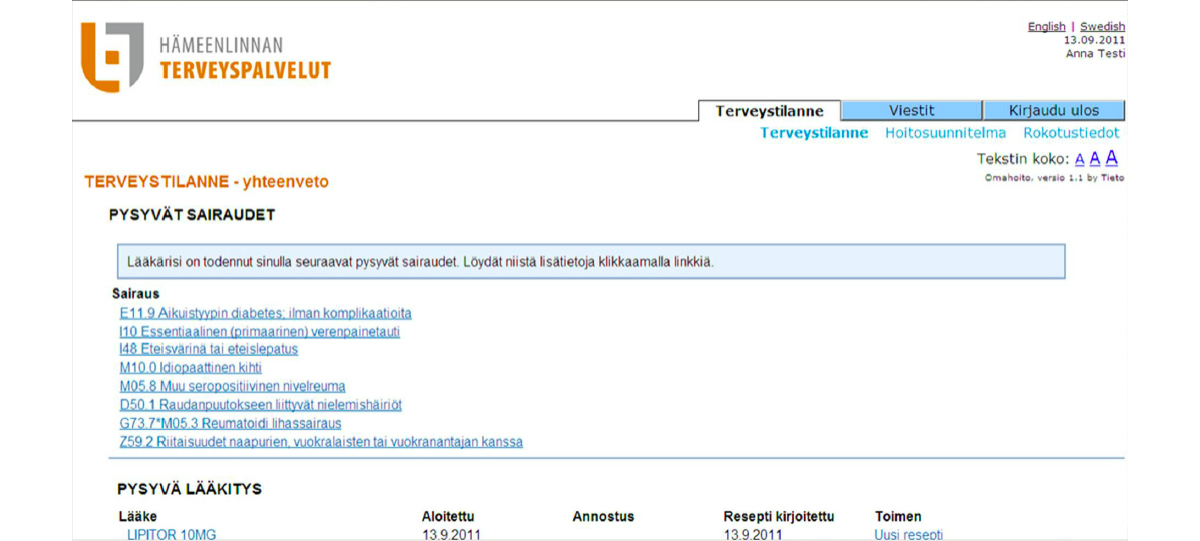
Screenshot of the patient portal.

The eligible patients were offered access to the patient portal during their visit to primary health care facilities (Figure 1). During the visit, they were given information on the contents and use of the portal. Patients could access the portal through the health care provider’s home page, using their bank identifiers for electronic identification. The functionalities of the portal included viewing the patient’s personal health record containing diagnoses, medication prescriptions, and laboratory results, viewing a personal care plan that the patient and a nurse had drawn up together during an in-person visit in order to holistically care for the patient’s health, electronic messaging with the care team, and prescription renewal.

Informed consent to participate was collected from each participant. Ethical approval was granted by the ethical board of the local authority (Pirkanmaa Hospital District).

### Materials

Owing to the diagnostic heterogeneity of the study group, three different types of diagnosis indicators were gathered to represent the comorbidity of the patients. First, diagnoses for the most common chronic illnesses in the study group, namely type 1 or 2 diabetes, hypertension, and hypercholesterolemia, were identified through the International Classification of Diseases (ICD-10) codes. Second, the Charlson Comorbidity Index (CCI) was used to assess the comorbidity of the patients. The CCI is a widely used system for characterizing patient comorbidities, drawing on ICD-10 recordings of 17 common chronic medical conditions (Multimedia Appendix 1) [10]. Third, the total number of diseases identified as chronic by a health care professional were collected from patients’ EHR.

The indicators for patients’ state of health were physiological health outcomes and health as reported by the patients. Values of the physiological measures glycated hemoglobin (HbA1c), low-density lipoprotein (LDL), body mass index (BMI), and blood pressure were collected to assess the patients’ state of physiological health. These measures were chosen because of their relevance in the management of the above-mentioned most common diseases in the participant group. The patients’ reported health was assessed through the short-form health survey SF-36, a broadly used instrument that generates functionality-based scores for mental and physical health and wellness [11-13].

The care received by the patients was obtained from the EHR databases. Using the unique personal ID code, the service contacts and the physiological outcomes measurement in the year preceding access to the portal were retrieved. Service contacts included doctor and nurse visits and calls in primary care and referrals to specialized care. To assess the monitoring, we collected the measurements of the patients’ relevant physiological health outcomes (HbA1c, BMI, LDL, and blood pressure).

To measure patient activation, we used the short form of Patient Activation Measure (PAM13), created by Judith Hibbard and colleagues [14], a validated instrument that assesses patient knowledge, skills, and confidence for self-management [14].

With the exception of SF-36 and PAM13 scores, all indicators were collected from the health care providers’ EHR. An email with a link to a survey was sent to participants to collect their responses to SF-36 and PAM surveys at the time of offering access to the portal, and 150 participants responded. The patient portal’s log information was collected to assess the use of the portal’s functionalities.

### Statistical Analysis

To analyze the predictors of patient portal use, participants were divided into three groups (non-users, viewers, and interactive users), based on their portal use during the 6 months after gaining access to the portal. Non-users did not log in to the portal during the follow-up period. Viewers logged in at least once, but did not use either of the interactional functionalities, namely messaging with the care team or prescription renewal. Interactive users logged in to the portal and used one of the interactional functionalities at least once.

For categorical variables, chi-square tests for overall differences among the three groups were used. To further identify such differences, pairwise comparisons using chi-square tests were conducted. Owing to the non-normality of the distributions for continuous variables, the non-parametric Kruskal-Wallis test was used to analyze overall differences among the three groups and the Wilcoxon-Mann-Whitney test for pairwise comparisons.

All statistical analyses were performed using Stata version 13 (StataCorp LP, College Station, TX). We used a CHARLSON Stata module by Stagg [15] to identify the CCI conditions from patient records and define the index value for each patient.

## Results

### Participants

A total of 876 patients visiting the health center facilities during the recruitment period from October 2011 to March 2012 fulfilled the eligibility criteria and were asked to participate in the study. Of these, 222 patients (25.3%) returned their informed consent and were included in the study. The mean age of patients was 62.7 (SD 9.0) years, and 49.1% (109/222) of them were women. The most frequent diagnoses of the study participants were type 1 or 2 diabetes, hypertension, and hypercholesterolemia. The majority of the patients had one disease or no diseases included in the CCI. The participants had visited a primary care doctor 3.4 (SD 3.2) times and a nurse 4.1 (SD 7.3) times, on average during the year before access to the portal (Table 1).

### Use of Patient Portal Functionalities

Once they logged in to the portal, patients would encounter the starting page, containing their own health information, including diagnoses, medication prescriptions, and laboratory results. On average, this information was viewed 17.0 (SD 20) times per patient during the first year after access to the portal. The second most popular viewed feature of the portal, used 4.5 (SD 6.0) times on average, was their own personal care plan. On average, patients sent 2.1 (SD 3.5) messages to their care team and viewed their vaccination record 1.6 (SD 1.9) times. Only 0.4 (SD 1.1) prescription renewals per patient were made through the portal during the first year after access (Table 2).

**Table 1:** Descriptive characteristics of the study participants.

Characteristic		Total (n=222)
Age, mean (SD)		62.7 (9.0)
Female		109 (49.1%)
**Most frequent diagnoses**		
	Type 1 or 2 diabetes^ab^	103 (46.4%)
	Hypertension^ac^	96 (43.2%)
	Hypercholesterolemia^ad^	139 (62.6%)
**Charlson comorbidity index** ^a^		
	0	93 (41.9%)
	1	79 (35.6%)
	2	50 (22.5%)
**Office visits** ^e^ **, mean (SD)**		
	Doctor visits	3.4 (3.2)
	Nurse visits	4.1 (7.3)

^a^From the time before access to the portal.

^b^ICD10 codes E10-E14 or ICPC codes T89-T90.

^c^ICD10 codes I10-I15 or ICPC codes K85-K87.

^d^ICD10 codes E78 or ICPC T93.

^e^During the year before access to the portal.

**Table 2:** Use of patient portal functionalities during the year after patient access.

Functionality	Mean (n=222)	SD	Min.	Max.
Viewing personal health record	17.0	20.0	0	146
Viewing personal care plan	4.5	6.0	0	44
Messages to the care team	2.1	3.5	0	25
Viewing vaccination record	1.6	1.9	0	13
Prescription renewal	0.4	1.1	0	7

### Characteristics Predicting Patient Portal Use

The overall differences in age, gender, diagnoses, health outcomes, received monitoring, service contacts, and patient activation among the three groups are presented in Table 3. Multiple pairwise comparisons for the same characteristics are presented in Multimedia Appendix 2.

The most significant differences between non-users, viewers, and interactive users were detected in monitoring and service contacts. The proportion of patients who had had their HbA1c measured and recorded during the year before access to the portal differed significantly between the groups (chi-square test, *P*=.03), being higher in the non-user group than among the viewers (pairwise chi-square test, *P*=.02) and the interactive user group (pairwise chi-square test, *P*=.01). Compared to the viewer group, the non-users were also more likely to have had their BMI (pairwise chi-square test, *P*=.02) and blood pressure (pairwise chi-square test, *P*=.02) measured and recorded. The non-users had visited a nurse most often (Kruskal-Wallis test, *P*=.01) but had fewer referrals to specialized care than the viewers (Wilcoxon-Mann-Whitney test, *P*=.02) and the interactive users (Wilcoxon-Mann-Whitney test, *P*=.03). The viewers and the interactive users differed from each other in the number of nurse calls. The interactive users had received more nurse calls during the preceding year than the viewers (Wilcoxon-Mann-Whitney test, *P*=.03).

The only comorbidity indicator that differed between the groups was the prevalence of hypertension diagnosis. The non-users were most likely to have a hypertension diagnosis (Kruskal-Wallis test, *P*=.01). There were no significant differences in mean age, gender distribution, or patient activation among the user profile groups. The statistically significant differences (*P*<.05) in patient characteristics among the user groups are presented in Table 4.

**Table 3:** Overall differences in patient characteristics among non-users, viewers, and interactive users.

Characteristic		Non-users (n=37)	Viewers (n=91)	Interactive users (n=94)	*P* value for difference among groups^h^
Female, n (%)		20 (54.1%)	44 (48.4%)	45 (47.9%)	.80
Age, years, mean (median, SD)		62.0 (64, 11.6)	63.0 (65, 8.8)	62.8 (65, 8.1)	.88
**Comorbidity, n (%)**					
	Type 1 or 2 diabetes^ab^	20 (54.1%)	36 (39.6%)	47 (50.0%)	.22
	Hypertension^ac^	24 (64.8%)	35 (38.5%)	37 (39.4%)	.01
	Hypercholesterolemia^ad^	22 (59.5%)	62 (68.1%)	55 (58.5%)	.37
**Charlson index, n (%)**					.51
	0	15 (40.5%)	40 (44.0%)	38 (40.4%)	
	1	10 (27.0%)	34 (37.4%)	35 (37.2%)	
	2	12 (32.4%)	17 (18.7%)	21 (22.3%)	
Chronic diagnoses, mean (median, SD)		2.3 (2, 1.5)	1.9 (2, 1.8)	1.9 (2, 1.8)	.22
**Physiological health outcomes, mean (median, SD)**					
	Glycated hemoglobin (HbA1c)^g^	42.4 (41.25, 9.4)	41.9 (39.8, 8.4)	44.2 (40.3, 12.7)	.96
	Low-density lipoprotein (LDL)^g^	2.7 (0.8)	3.0 (1.0)	3.0 (1.0)	.25
	Body mass index (BMI)^g^	31.0 (32.0, 5.6)	31.7 (31.0, 6.7)	31.5 (30.0, 6.8)	.98
	Blood pressure, diastolic^g^	82.2 (84.5, 11.8)	86.5 (87.5, 10.5)	86.4 (86.5, 10.6)	.17
	Blood pressure, systolic^g^	139.7 (140.3, 16.3)	143.7 (142.0, 21.4)	144.1 (145.0, 17.7)	.50
**Patient-reported health, mean (median, SD)**					
	SF-36 Physical Health at access	63.2 (66.0, 22.1)	65.9 (69.0, 20.4)	63.5 (66.5, 20.6)	.77
	SF-36 Mental Health at access	75.9 (80.0, 16.3)	75.8 (80.0, 19.0)	71.0 (78.5, 22.6)	.59
**Monitoring, n (%)**					
	At least one HbA1c measurement^e^	31 (83.8%)	57 (62.6%)	57 (60.6%)	.03
	At least one LDL measurement^e^	32 (2.48, 86.5%)	74 (2.83, 81.3%)	77 (2.81, 81.9%)	.77
	At least one BMI measurement^e^	31 (83.8%)	57 (62.6%)	66 (70.2%)	.06
	At least one blood pressure measurement^e^	34 (91.9%)	67 (73.63%)	77 (81.9%)	.05
**Service contacts, mean (median, SD)**					
	Doctor visits ^e^	3.4 (2, 3.5)	2.9 (2, 2.6)	3.9 (3, 3.7)	.27
	Nurse visits^e^	5.2 (4, 3.9)	3.7 (3, 3.1)	4.7 (3, 10.8)	.01
	Doctor calls^e^	1.1 (0, 1.6)	1.3 (1, 1.6)	1.5 (1, 1.8)	.50
	Nurse calls^e^	1.0 (0, 1.3)	0.8 (0, 1.4)	1.0 (1, 1.7)	.13
	Referrals to secondary care^e^	0.2 (0, 0.7)	0.4 (0, 0.6)	0.5 (0, 0.8)	.15
**Patient activation (PAM), mean (median, SD)**					
	PAM score at access	63.5 (63.2, 11.7)	63.8 (66.0, 15.5)	62.4 (63.2, 15.1)	.84

^a^From the time before access to patient portal.

^b^ICD10 codes E10-E14 or ICPC codes T89-T90.

^c^ICD10 codes I10-I15 or ICPC codes K85-K87.

^d^ICD10 codes E78 or ICPC T93.

^e^During the year before access to patient portal.

^g^At least one measurement during the year before access to patient portal. If a patient had several measurements, the average is reported.

^h^Chi-square test for the categorical variables and non-parametric Kruskal-Wallis test for the continuous variables.

**Table 4:** Differences in patient characteristics between user groups.

Characteristics	Non-users	Viewers	Interactive users
Comorbidity	Most likely to have a hypertension diagnosis		
Monitoring	Most likely to have had their HbA1c measured and recorded		
	More likely than viewers to have had their BMI and BP measured and recorded	Less likely than non-users to have had their BMI and BP measured and recorded	
Service contacts	Most nurse visits		
	Least referrals to specialized care	Fewer nurse calls than interactive users	More nurse calls than viewers

## Discussion

### Principal Findings

In this study, we analyzed how patients’ state of health, previous care received, age, gender, and patient activation predict the use of an electronic patient portal. The differences in these indicators were assessed between non-users, viewers, and interactive users. The main differences were detected in the previous care received by non-users and the two user groups that logged in to the portal.

Previous research has found positive [16-18] and negative [19,20] associations between patient portal use and use of other health care services. In this study, this association was found to vary by the type of health services previously received. Whereas the non-user group had visited a nurse most often, they had fewer referrals to specialized care than the two groups that had logged in to the portal. Among the patients who had logged in, the interactive users differed from the viewers in having received more nurse calls during the year before access. Plausible explanations for these observations may be found by considering the patient needs that an electronic patient portal potentially meets. In Finland, routine monitoring of the chronically ill is mostly performed by nurses. A lack of referrals to specialized care, combined with a higher number of nurse visits, may indicate a stable medical condition where patient needs are met and new channels for medical services are not needed. The association between higher numbers of nurse calls and interactive use of a patient portal may be explained by the nature of the interaction performed through these service channels. Compared to face-to-face visits with a health care provider, service encounters conducted by phone may be more apt for substitution by online interaction. Whereas these possible explanations are just some of many alternatives, the findings of this study do encourage, in line with Varsi and colleagues [21], a more fine-grained distinction between different types of health service encounters and respective patient-provider communication channels.

Unlike previous service use, state of health, age, gender, and patient activation had no significant association with patient portal use in this study. Previous findings on these associations are somewhat contradictory. In a study by Weingart and colleagues [19], patients who enrolled in a patient portal had fewer medical problems, and Tenforde and colleagues [22] found, among a diabetic cohort, that the users of a patient portal demonstrated better glycemic control. By contrast, the study by Earnest and colleagues [16] showed that the portal users were more symptomatic than the non-users. On the association between portal use and patient activation, Hibbard and Greene [23] stated that more activated patients are more likely to be referred to the patient portal and, among that group, the higher activated were more likely to actually use it, whereas Roblin and colleagues [24] found no significant association. In previous studies, younger [19,22] or older [18], and men [25] or women [24] have been suggested to be more likely or frequent users of patient portals.

The heterogeneity of the research results is likely due to the different settings of the studies. In particular, the chosen cohort and the functionalities offered through the portal may yield differing results. In this study, the participant group consisted of chronically ill patients, who were thus likely to benefit from the portal and were all explicitly offered access to it. Further, the portal itself included little functionality or information that might not be accessed at all via the traditional service channels, namely in-person visits and phone calls. Thus, it is not surprising that age, gender, patient activation, and state of health lost their relevance, whereas patients’ demand for care and preferences in terms of service channel came to matter in the use of the patient portal. It should be noted, however, that only a minority of the study participants never logged in to the portal. This finding supports the suggestion that chronically ill patients are likely to benefit from, and thus to use, an electronic patient portal.

Some of the findings on predictors of patient portal use may also have reflected the contemporary novelty of health services delivered through the Internet. Lately, it has been suggested that the digital divide between different sociodemographic groups due to lack of access to the Internet is narrowing [26]. In Finland in 2013, 92% of people aged 16 to 74 years and 85% of people aged 16 to 89 years had used the Internet in the past 3 months. Further, 79% of people aged 16 to 89 years had used Internet banking in the past 3 months [27]. It is thus unlikely that access to the Internet or the novelty of running errands online would, in general, hamper access to electronic patient portals, although health care-related online services are still something of a novelty in Finland. Rather, the results suggest that the non-user group did not perceive an additional benefit in using the portal, as they were already well served or they preferred the traditional service channels to electronic services. An important aspect of patient portal as a service channel is that, through it, receiving and providing information is not tied to time and place. To assess the value of this aspect to the patient, future research may benefit from applying behavioral research [28] or economic models on the individual demand for health services or health information in general [29].

In addition to the patient cohort targeted and the patient needs that are met through the portal, future research should pay attention to the influence of the personnel marketing the portal and its functionalities to the patients. In this study, the portal was explicitly offered to each participant, so the choice of use was left to the patient. Nevertheless, it is possible that, for example, some of the health centers involved in the study were encouraging use of the portal more than others. Further, while the prescription renewal functionality was offered in the portal, its use was not promoted, owing to the lack of a national prescriber-pharmacist interface at the time of the study.

### Strengths and Limitations

This study contributes to previous research by extracting predictors for patients’ choice to use an electronic patient portal in a group of patients that is likely to benefit from such portal, namely the chronically ill. Unlike in most studies with large patient cohorts [19], the portal was explicitly offered to each patient. The views of staff in the health care service on who may benefit from the portal did not therefore affect subsequent categorization of patients among user and non-user groups. Further, when analyzing the association between portal use and other services received, different contact types were specified. This provided a more refined view, typically neglected by previous studies [20]. Patients’ state of health, which has an apparent connection with patients’ need of and demand [30] for health care services, was assessed by several objective and subjective measures. This supports the validity of the conclusion that the groups did not differ in terms of disease burden but rather in their demand for different services or preference for different service channels.

Despite these contributions, the study also has limitations. A major and common limitation is the restriction of the study results to the patients who responded to the study request and who may fundamentally differ from those who decided not to participate. Further, the empiria of this study does not provide evidence of *why* the use of certain services is related to use of a patient portal. Neither was the effect of patient portal use on relevant care outcomes addressed. Further studies, analyzing different customer relationships and the relevance of a patient portal in these relationships, are needed to gain understanding on which functionalities of a patient portal may complement or substitute for traditional channels for service delivery in health care, and to assess the effect that this substitution may have on care outcomes.

### Conclusions

In this study, the predictors of the use of an electronic patient portal were assessed among a group of patients likely to benefit from such a portal, namely the chronically ill. Previous care received by the patient, rather than state of health, age, gender, and patient activation, was an important factor predicting the attractiveness of electronic patient portal use.

Previous research on patient characteristics predicting the use of electronic patient portals has shown contradictory results. This is partially due to the differences in patient cohorts and portal functionalities. However, some of the predictors may also be losing their relevance as the novelty of online health care services levels out. As sociodemographic factors become less accurate predictors of online service use in Western countries, individual preferences in terms of service channel, as well as the functionalities offered through a patient portal, become relevant when identifying the potential uses of such a portal. To grasp the full potential of electronic patient portals, care providers need to know what types of services may be provided through a patient portal. Further research on patient portal functionalities and their potential to meet patient needs by complementing or replacing traditional health care services is suggested.

## Multimedia Appendix 1

Charlson Comorbidity Index conditions.

## Multimedia Appendix 2

Pairwise comparisons of patient characteristics.

## Abbreviations

BMI: body mass index
CCI: Charlson comorbidity index
EHR: electronic health record
HbA1c: glycated hemoglobin
LDL: low-density lipoprotein
PAM: patient activation measure
