# Supplementary material for: Use of an Electronic Patient Portal Among the Chronically Ill: An Observational Study
Source: J Med Internet Res. 2014 Dec 8;16(12):e275. doi: 10.2196/jmir.3722 (PMC4275506; doi:10.2196/jmir.3722)
Supplement: Supplementary file 1 [file jmir_v16i12e275_app1.pdf]

Multimedia Appendix 1. Charlson Comorbidity Index conditions.

Myocardial infarction
Congestive heart failure
Peripheral disease
Cerebrovascular disease
Dementia
Chronic pulmonary disease
Connective tissue disease
Peptic ulcer disease
Mild liver disease
Hemiplegia
Moderate or severe renal disease
Diabetes with end-organ damage
Tumor
Leukemia
Lymphoma
Moderate or severe liver disease
AIDS
