# Supplementary material for: Use of an Electronic Patient Portal Among the Chronically Ill: An Observational Study
Source: J Med Internet Res. 2014 Dec 8;16(12):e275. doi: 10.2196/jmir.3722 (PMC4275506; doi:10.2196/jmir.3722)
Supplement: Supplementary file 2 [file jmir_v16i12e275_app2.pdf]

Multimedia Appendix 2. Pairwise comparisons of patient characteristics.

Characteristic	Non-users (n=37)	Viewers (n=91)	Interactive Users (n=94)	P-value for pairwise group differences		
				Non-users vs. viewes	Non-users vs. Interactive users	Viewers vs. Interactive users
Female (n, %)	20 (54.1)	44 (48.4)	45 (47.9)	.56	.52	.95
Age, mean (Mdn, SD)	62.0 (64, 11.6)	63.0 (65, 8.8)	62.8 (65, 8.1)	.64	.67	.91
**Comorbidity**						
Type 1 or 2 diabetes[ab] (n, %)	20 (54.1)	36 (39.6)	47 (50.0)	.13	.67	.15
Hypertension[ac] (n,%)	24 (64.8)	35 (38.5)	37 (39.4)	.01	.01	.90
Hypercholesterolemia[ad] (n, %)	22 (59.5)	62 (68.1)	55 (58.5)	.35	.92	.18
Charlson index				.21	.39	.80
0 (n, %)	15 (40.5)	40 (44.0)	38 (40.4)			
1 (n, %)	10 (27.0)	34 (37.4)	35 (37.2)			
2 (n, %)	12 (32.4)	17 (18.7)	21 (22.3)			
Chronic diagnoses, mean (Mdn, SD)	2.3 (2, 1.5)	1.9 (2, 1.8)	1.9 (2, 1.8)	.11	.09	>.99
**Physiological health Outcomes**						
HbA1c [g], mean (Mdn, SD)	42.4 (41.25, 9.4)	41.9 (39.8, 8.4)	44.2 (40.3, 12.7)	.83	.90	.81
LDL [g], mean (Mdn, SD)	2.7 (0.8)	3.0 (1.0)	3.0 (1.0)	.15	.10	.83
BMI [g], mean (Mdn, SD)	31.0 (32.0, 5.6)	31.7 (31.0, 6.7)	31.5 (30.0, 6.8)	.83	.88	.88
Blood pressure, diastolic [g], mean (Mdn, SD)	82.2 (84.5, 11.8)	86.5 (87.5, 10.5)	86.4 (86.5, 10.6)	.06	.11	.82
Blood pressure, systolic [g], mean (Mdn, SD)	139.7 (140.3, 16.3)	143.7 (142.0, 21.4)	144.1 (145.0, 17.7)	.54	.24	.54
**Patient-reported health**						
SF-36 Physical Health at access, mean (Mdn, SD)	63.2 (66.0, 22.1)	65.9 (69.0, 20.4)	63.5 (66.5, 20.6)	.64	.86	.50
SF-36 Mental Health at access, mean (Mdn, SD)	75.9 (80.0, 16.3)	75.8 (80.0, 19.0)	71.0 (78.5, 22.6)	.81	.54	.33
**Monitoring**						
At least one HbA1c measurement[e] (n, %)	31 (83.8)	57 (62.6)	57 (60.6)	.02	.01	.78
At least one LDL measurement[e] (n, %)	32 (2.48, 86.5)	74 (2.83, 81.3)	77 (2.81, 81.9)	.48	.53	.92
At least one BMI measurement[e] (n, %)	31 (83.8)	57 (62.6)	66 (70.2)	.02	.11	.28

At least one blood pressure measurement[e] (n, %)	34 (91.9)	67 (73.63)	77 (81.9)	.02	.15	.18
**Service contacts**						
Doctor visits [e], mean (Mdn, SD)	3.4 (2, 3.5)	2.9 (2, 2.6)	3.9 (3, 3.7)	.53	.55	.10
Nurse visits [e], mean (Mdn, SD)	5.2 (4, 3.9)	3.7 (3, 3.1)	4.7 (3, 10.8)	<.01	.01	.82
Doctor calls [e], mean (Mdn, SD)	1.1 (0, 1.6)	1.3 (1, 1.6)	1.5 (1, 1.8)	.29	.23	.79
Nurse calls [e], mean (Mdn, SD)	1.0 (0, 1.3)	0.8 (0, 1.4)	1.0 (1, 1.7)	.24	.68	.03
Referrals to secondary care[e], mean (Mdn, SD)	0.2 (0, 0.7)	0.4 (0, 0.6)	0.5 (0, 0.8)	.02	.03	.83
**Patient activation**						
PAM score at access, mean (Mdn, SD)	63.5 (63.2, 11.7)	63.8 (66.0, 15.5)	62.4 (63.2, 15.1)	.81	.70	.59
